# Environmental cadmium exposure alters the internal microbiota and metabolome of Sprague–Dawley rats

**DOI:** 10.3389/fvets.2023.1219729

**Published:** 2023-07-26

**Authors:** Songqing Liu, Xin Deng, Zheng Li, Wenjing Zhou, Gang Wang, Jiasui Zhan, Binhong Hu

**Affiliations:** ^1^College of Chemistry and Life Sciences, Chengdu Normal University, Chengdu, China; ^2^Sichuan Provincial Key Laboratory for Development and Utilization of Characteristic Horticultural Biological Resources, Chengdu Normal University, Chengdu, China; ^3^North Sichuan Medical College, Nanchong, China; ^4^Department of Forest Mycology and Plant Pathology, Swedish University of Agricultural Sciences, Uppsala, Sweden

**Keywords:** cadmium exposure, *16S rRNA*, blood microbiome, serum metabolism, SD rats

## Abstract

Cadmium (Cd) is a toxic element that can negatively affect both humans and animals. It enters the human and animal bodies through the respiratory and digestive tracts, following which it tends to accumulate in different organs, thereby seriously affecting human and animal health, as well as hampering social and economic development. Cd exposure can alter the composition of intestinal microbiota. In addition, it can damage the peripheral organs by causing the translocation of intestinal microbiota. However, the relationship between translocation-induced changes in the composition of microbiome in the blood and metabolic changes remains unclear. In the present study, we investigated the effects of Cd exposure on microbiota and serum metabolism in rats by omics analysis. The results demonstrated that Cd exposure disrupted the balance between the blood and intestinal flora in Sprague–Dawley (SD) rats, with a significant increase in gut microbiota (*Clostridia_UCG_014*, *NK4A214_group*) and blood microbiome (*Corynebacterium*, *Muribaculaceae*). However, Cd exposure caused the translocation of *Corynebacterium* and *Muribaculaceae* from the gut into the blood. In addition, Cd exposure was associated with the up-regulation of serum indoxyl sulfate, phenyl sulfate, and p-cresol sulfate; down-regulation of δ-tocopherol and L-glutamine; and changes in blood microbiome and metabolites. In conclusion, we identified novel metabolic biomarkers for Cd toxicity, which will also expand our understanding of the role of blood microbiome in Cd-induced injury.

## 1. Introduction

The pollution caused by heavy metals and their impact on the environment has been increasing, with their effects extending to different environmental compartments such as water, soil, and atmosphere (1, 2). Among the 17 prominent food contaminants identified by the World Health Organization (WHO), cadmium (Cd) occupies the third position, after aflatoxin and arsenic. Cd is a highly toxic and accumulative environmental pollutant with strong chemical activity, persistent toxicity, and high soil mobility. It is easily absorbed and transported via plant roots into the plant body, where it accumulates in the edible parts (3, 4). In addition, bioaccumulation of Cd in animals and humans has been reported, along the food chain, resulting in detrimental health effects (2) and perpetuating a persistent cycle of pollution. Excessive exposure to Cd has been reported to trigger oxidative stress, induce cellular and molecular alterations that disrupt the body’s metabolic equilibrium, and damage the intestine, resulting in dysbiosis (2, 5).

The gut microbiota serves as a crucial mediator of Cd-induced toxicity and is intricately involved in host toxicology. Exposure to Cd increases the abundance of pathogenic intestinal bacteria and disrupts the microbial ecosystem, leading to microecological dysbiosis (5, 6). Changes in the composition of the gut microbiome can affect the pathology of peripheral organs in several ways (7), transfer functional biomolecules horizontally between bacterial and mammalian host cells by releasing the outer membrane vesicles (OMVs) (7), and affect the levels of short-chain fatty acids (SCFAs) by regulating energy metabolism (8). SCFAs are known to participate in Cd-induced intestinal inflammation and metabolic disturbance (9). In addition, the translocation of gut microbes from the gut to the blood circulation has been demonstrated by previous studies (10). Increased intestinal permeability has been known to contribute to bacterial translocation, a process wherein detrimental bacteria traverse the intestinal barrier and gain access to the bloodstream, thereby increasing the susceptibility of the body to infections (11–13).

Blood microbiome has been a recent discovery, and specific bacterial species in blood have been reported to be associated with several infections and inflammation-related diseases (11, 12, 14). For example, an increased abundance of *Sediminibacterium* in the blood is associated with a heightened risk of developing type 2 diabetes mellitus (T2DM) (15). Increased abundances of Proteobacteria phylum, Gammaproteobacteria class, and Enterobacteriaceae and Pseudomonadaceae families were found in the blood of patients with chronic kidney disease (CKD) (16). The primary source of blood microbiome has been reported to be microorganisms transferred from the gut or oral cavity (11, 12). Cd exposure is known to trigger inflammation and disrupt intestinal permeability, allowing microbes to enter the blood circulation and inducing metabolic disorders (13, 17, 18). For example, serum metabolomics revealed a pathway by which Cd exposure disrupts lipid and amino acid metabolism (19). In addition, Cd exposure increases the risk of metabolic diseases including cardiovascular diseases and metabolic syndromes (20, 21). Therefore, identifying novel metabolomic biomarkers will lead to the development of effective strategies for heavy metal detoxification.

Our current knowledge of the impact of Cd exposure on the composition of microbes in the gastrointestinal tract and bloodstream, as well as its effect on serum metabolites, is limited. In the present study, we investigated the association between changes in microbial abundance and serum metabolites in response to Cd exposure. The primary aim was to gain a deeper understanding of the relationship between microbiome and relevant metabolites in the context of health risks associated with Cd pollution.

## 2. Materials and methods

### 2.1. Establishment of the animal model

Sprague–Dawley (SD) female rats (6–8 weeks old and weighing 180–210 g) were purchased from Chengdu Dossy Experimental Animals Co., Ltd. The rats were placed in a controlled environment (temperature, 25 ± 3°C; relative humidity, 50 ± 5%), provided sufficient food and water, and treated with a 12 h/12 h light/dark cycle. The animals are kept in relatively sterile environment. All laboratory procedures were carried out in accordance with the guidelines and regulations of Animal Research Reporting of *In Vivo* Experiments (ARRIVE). The Ethics Committee of the Chengdu Normal University (grant no: CNDC-20210912034R) approved the disposal of animals during the experiment, which complied with the animal ethics standards. After 7 days of adaptation, the rats were randomly divided into two groups (*n* = 12 in each group): the CdCl_2_ group and the control group. Rats in the Cd-exposed groups were administered CdCl_2_ by gastric infusion at a daily dose of 5 mg/kg (22) body weight. The chemicals were prepared daily by dissolving them in distilled water. Twelve rats in the control groups received an equal volume of normal saline by regular gavage. The experiments were conducted for 30 days, due to the long-term deposition of Cd in the body. Therefore, we adopted the experimental model with a long observation time after exposure. Serum was collected from the tail vein. The rats were stunned with ether before being killed by neck dislocation, and the colorectum and feces were immediately harvested. Afterward, the samples were rinsed with ice-cold saline and stored at −80°C until further use.

### 2.2. Inflammatory factors and intestinal tight junction protein expression

We measured the levels of tumor necrosis factor (TNF)-α and interleukin (IL)-6 in the serum and changes in the content of ZO-1, TNF-α, and IL-6 in the colorectum of rats using an enzyme-linked immunosorbent assay (ELISA) kit according to the manufacturer’s instructions (Hepeng Biotechnology Co., Ltd., Shanghai, China).

### 2.3. DNA extraction and library construction

The total bacterial DNA was extracted from colorectal lumen contents and serum using the Qubit dsDNA Assay Kit (Life Technologies, United States). The DNA concentration and integrity were determined by spectrophotometry and agarose gel electrophoresis, respectively. We selected V3–V4 hypervariable regions based on the sequencing results (primers: 343F: 5′-TACGGRAGGCAGCAG-3′, 798R: 5′-AGGGTATCTAATCCT-3′) (23). The polymerase chain reaction (PCR) products were purified with Agencourt AMPure XP beads (Beckman Coulter Co., United States) and the Qubit dsDNA Assay kit (Thermo Fisher Scientific, United States), was used to quantify the final amplification. Sequencing was performed on an Illumina NovaSeq 6000 instrument with 250 bp paired-end reads.

### 2.4. Analysis of blood and intestinal microbiota

We used the Cutadapt software to cut the primer sequences. Raw data were subjected to quality control analysis according to the default parameters of QIIME 2 (24) to obtain representative sequences. The representative reads of each amplicon sequence variant (ASV) were selected using the QIIME 2 package (24). All representative reads were annotated and blasted against the Silva database version 138 using an Ribosomal Database Project (RDP) classifier (25) with a 70% confidence threshold.

### 2.5. Metabolomics

We accurately took 200 μL of serum in a 1.5 mL Eppendorf tube, to which 450 μL of precipitant protein methanol–acetonitrile (V:V = 2:1，containing 2-chloral-phenylalanine, 2 μg/mL) was added. This solution was vortexed for 1 min, followed by ultrasonic extraction for 10 min. After centrifugation for 10 min (13,000 g, 4°C), 150 μL of the supernatant was extracted and stored at −80°C until liquid chromatography-mass spectrometry (LC-MS) analysis and treatment. An ACQUITY UHPLC system (Waters Corporation; Milford, United States) coupled with an AB SCIEX Triple TOF 5600 System (AB SCIEX, Framingham, MA) was used to analyze the metabolic profiling in both electrospray ionization (ESI)-positive and ESI-negative ion modes. An ACQUITY UPLC BEH C18 column (1.7 μm, 2.1 × 100 mm) was used in both positive and negative modes. The binary gradient elution system consisted of A-water (0.1% formic acid, v/v) and B-acetonitrile (0.1% formic acid, v/v). The flowrate, column temperature and injection volume used in the system were 0.35 mL/min, 45°C and 2 μL, respectively. The data were acquired in the full-scan mode (*m*/*z* ranges from 70 to 1,000) in conjunction with the information dependent acquisition (IDA) mode.

### 2.6. Statistical analysis

For 16S and metabolomic analyses, multivariate statistical analyses [principal coordinate analysis (PCoA) and orthogonal partial least squares discriminant analysis (OPLS-DA)] and univariate statistical analyses (Wilcoxon rank-sum test, *t*-test, and correlation analysis) were performed extensively. For omics association analysis, Pearson’s or Spearman’s correlation was used to analyze the relationship between microbiota and metabolites. In addition, relationships between different bacterial flora and metabolites were analyzed. The R language and Cytoscape software were used to analyze the matrix heatmap and association network. Statistical methods were determined based on the data and project objectives. All data are expressed as mean ± standard deviation (SD). The Wilcoxon rank sum test or *t*-test was used for comparisons between the groups (^*^p < 0.05, ^**^p < 0.01, ^***^p < 0.001). GraphPad Prism 8.0 and SPSS 19.0 software were used for statistical analysis of variance and the significance tests.

## 3. Results

### 3.1. Effects of CdCl_2_ on intestinal tight junction proteins, and intestinal and blood inflammatory factors

**Figure 1:**
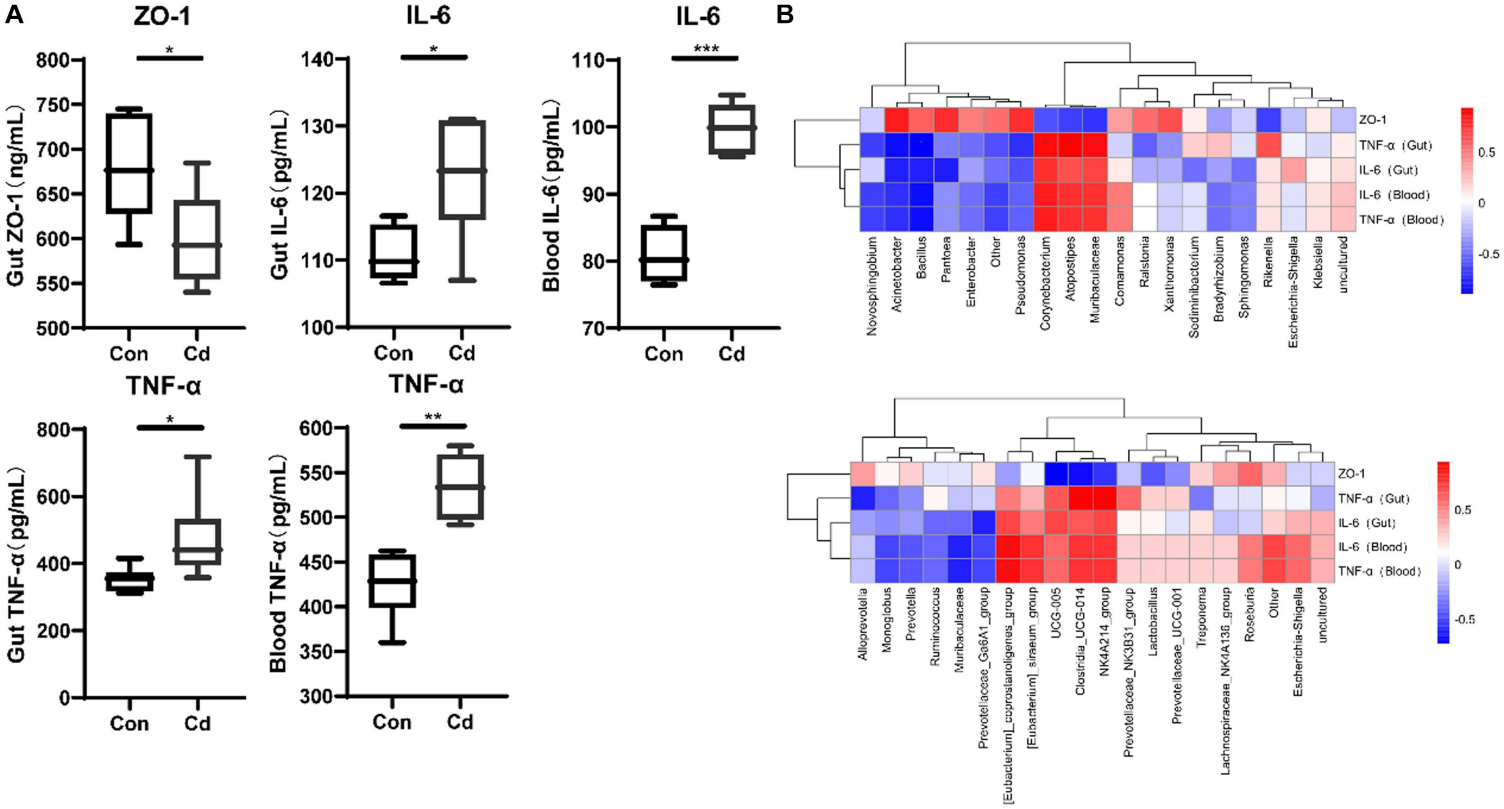
Effects of CdCl_2_ on intestinal tight junction protein, intestinal and blood inflammatory factors. **(A)** Ekxpression of ZO-1, TNF-α, and IL-6. All experimental data represent six independent experiments with similar results. **(B)** Correlation between ZO-1, inflammatory factors, and intestinal and blood microbiome.

Exposure to Cd significantly reduced the expression of ZO-1 protein in the intestine (*p* < 0.05). However, the levels of inflammatory factors TNF-α and IL-6 in the intestine and blood were significantly increased (*p* < 0.05) (Figure 1A). Spearman’s analysis showed that the expression of the *Clostridia_UCG_014*, *NK4A214_group*, *Corynebacterium*, *Muribaculaceae*, and *Atopostipes* was positively correlated with that of TNF-α in the intestinal tract (Figure 1B).

### 3.2. Cadmium exposure changed the relative abundance of blood and intestinal microbiome

**Figure 2:**
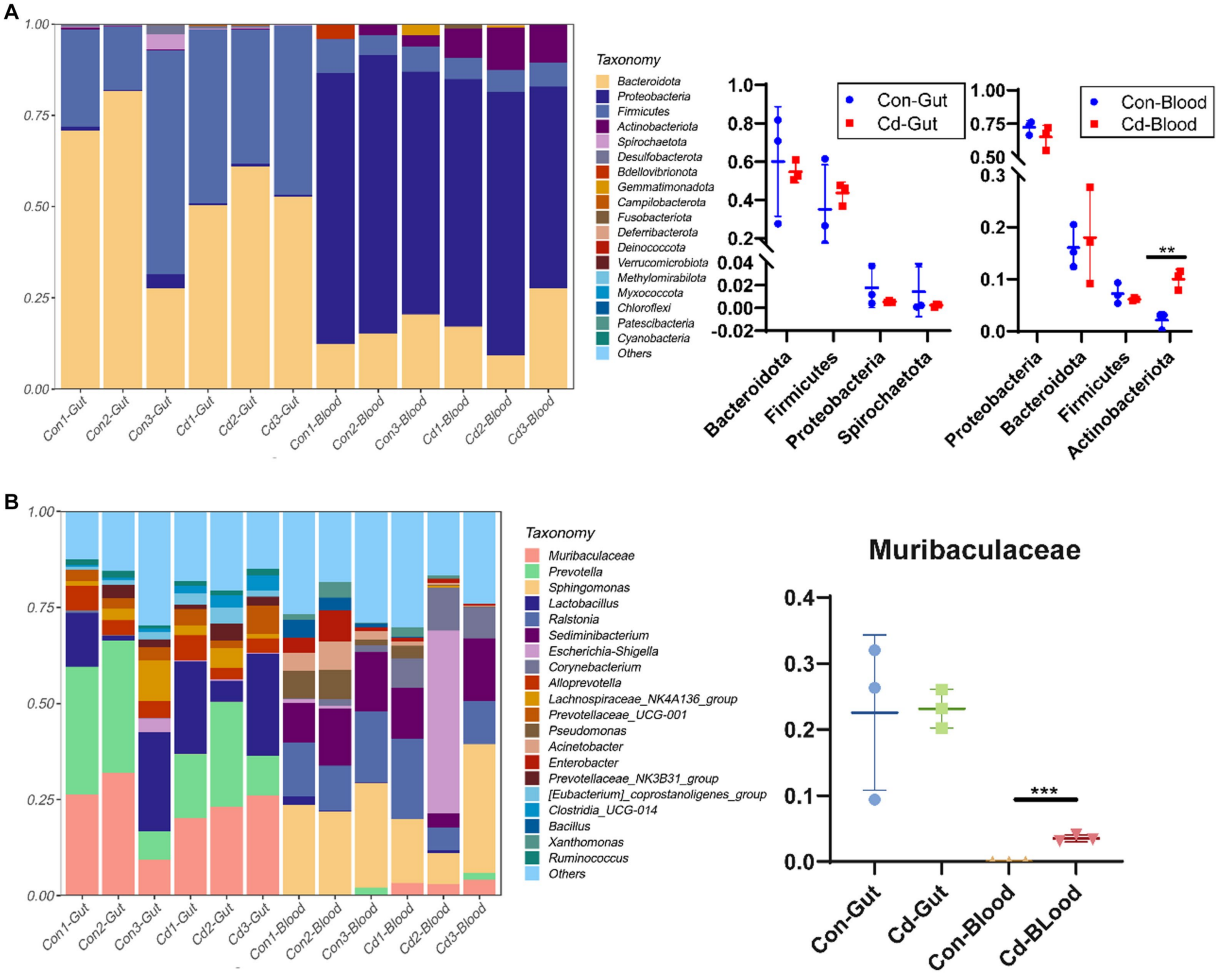
Relative abundance map of gut and blood microbiome. **(A)** Bar plot of the relative abundance of species at the phylum level. **(B)** Bar plot of the relative abundance at the genus level. ^*^Control, Con; CdCl_2_, Cd. All experimental data are representative of three independent experiments with similar results.

The number of valid tags obtained from clean tags after removing chimeras ranged from 21,105 to 70,194. The number of ASVs in each sample ranged from 53 to 381. The relative abundance of the top 20 ASVs was classified at the phylum and genus levels. Bacteroidota and Firmicutes were the major phyla in the intestinal tract.At the genus level, *Muribaculaceae* (22.84%), *Prevotella* (21.54%), *Lactobacillus* (16.17%), *and Alloprevotella* (4.67%) were the primary microorganisms. Similarly, Proteobacteria and Bacteroidota were the main microorganisms in the blood. At the genus level, *Sphingomonas* (21.82%), *Ralstonia* (13.69%), *Sediminibacterium* (12.30%), and *Escherichia* (8.23%) were the primary bacteria. Cadmium chloride increased the abundance of Actinobacteriota in the blood microbiome (*p* < 0.05, Figure 2A). *Muribaculaceae* was not detected in the normal blood, but in the cadmium-exposed group, its abundance increased significantly in the Cd-exposed group (Figure 2B).

### 3.3. Effects of cadmium exposure on microbial diversity in blood and gut

**Figure 3:**
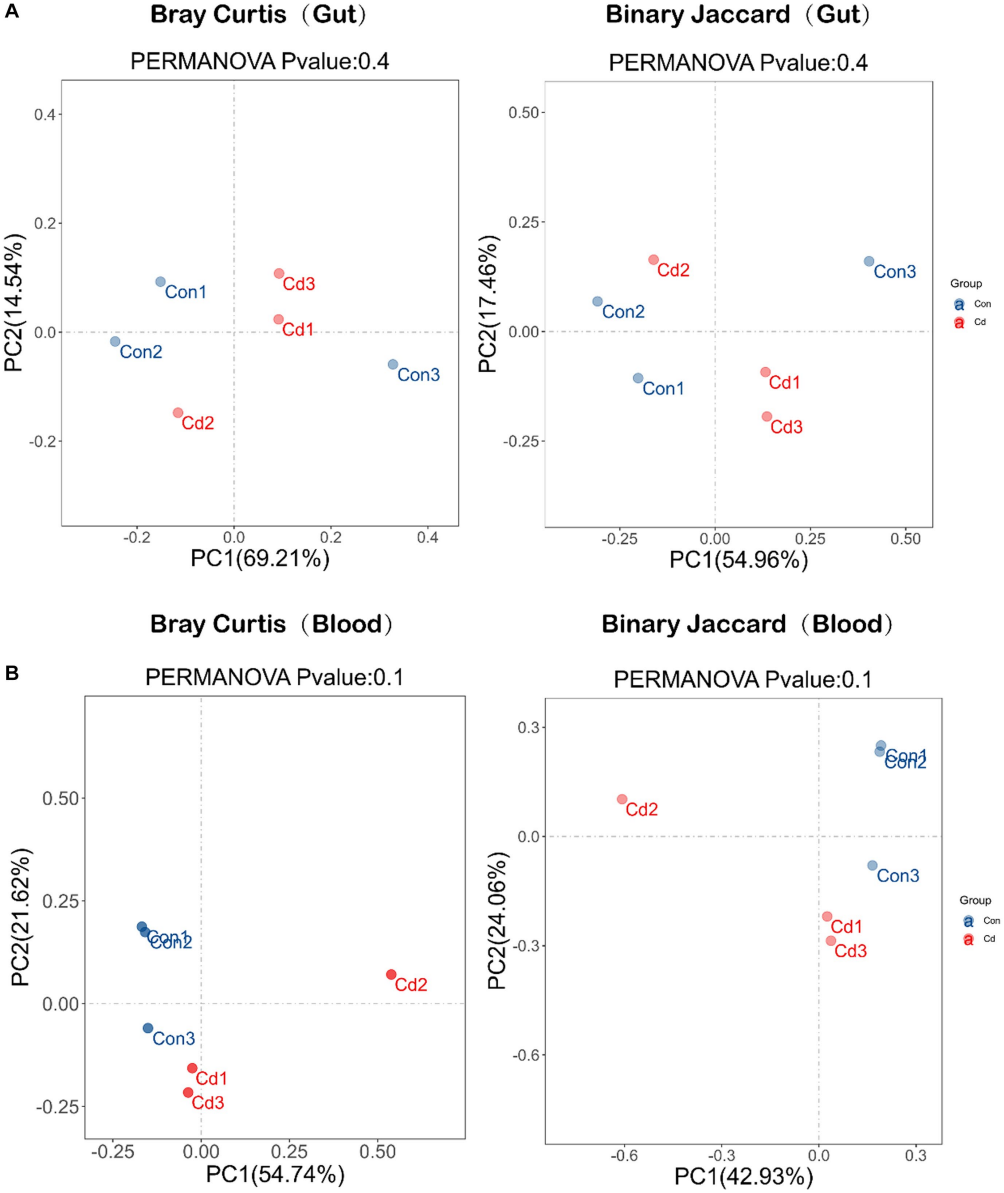
Diversity of microbial community composition: β-diversity was analyzed based on PCoA (principal coordinates analysis) and divided into Bray Curtis and Binary Jaccard. **(A)** β-diversity analysis of intestinal flora. **(B)** β-diversity analysis of blood flora.

Next, changes in the composition of intestinal and blood microbiome caused by Cd exposure were detected. However, the PCoA plots revealed no significant differences in the microbial composition of the gut and blood between the control and CdCl_2_ groups (Figure 3).

### 3.4. Effects of cadmium chloride on differential characteristic groups of blood and gut microbiome abundance

Linear discriminant analysis effect size (LEfSe) indicated species with significantly different abundance between the control and Cd-exposed group.

**Figure 4:**
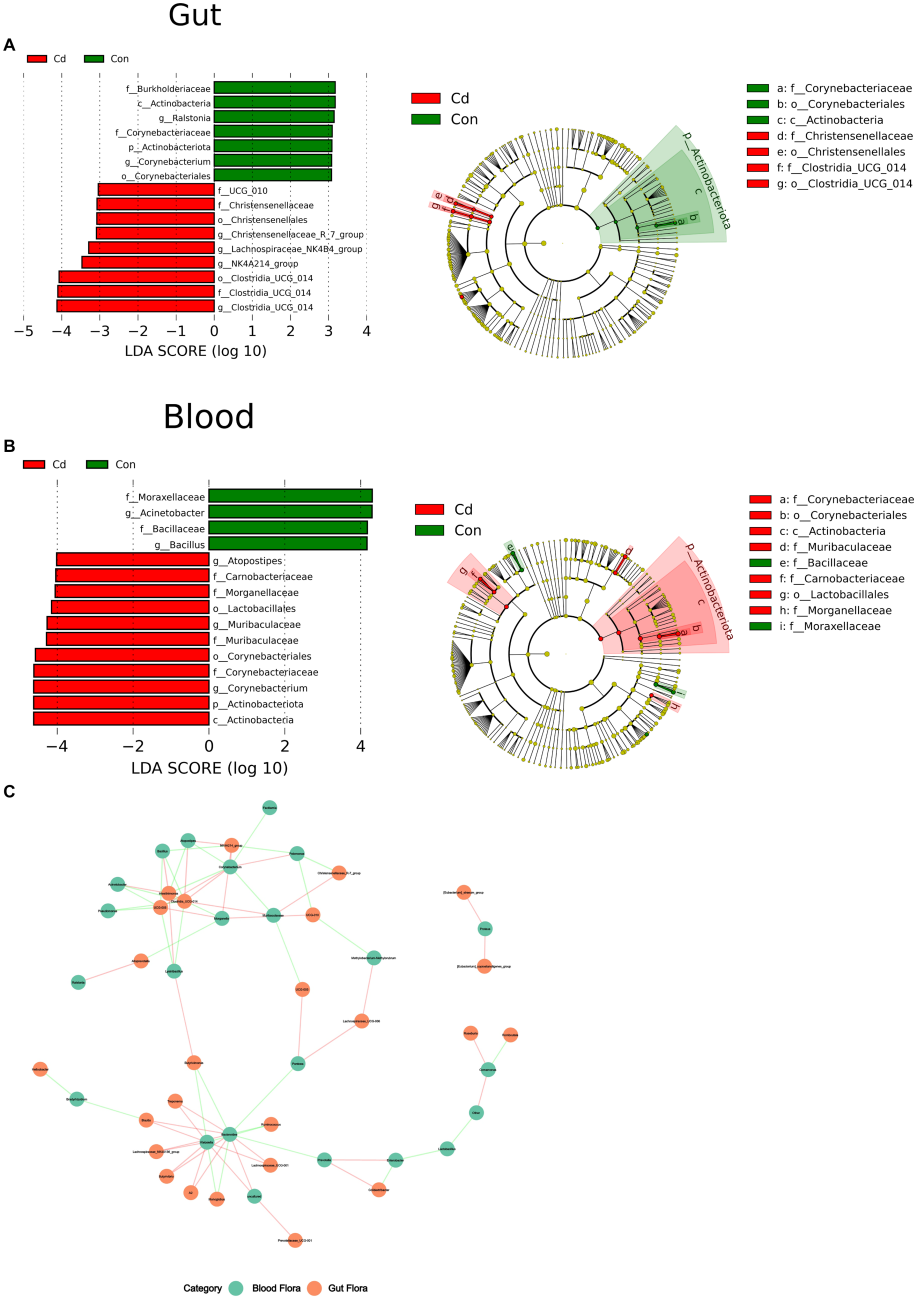
The Wilcoxon rank sum test for species differences between intestinal and blood microbiome. **(A)** The dominant species in the gut after Cd exposure; **(B)** Dominant species after Cd exposure in blood. **(C)** Correlation network analysis of the flora at the level of the genus in the gut and blood.

At the phylum level, the abundance of Acinetobacteriota in the CdCl_2_ group significantly decreased in the gut and increased in the blood. In the intestine, at the genus level, *Corynebacterium* and *Ralstonia* were the dominant flora in the control group. *Clostridia_UCG_014*, *NK4A214_group*, *Lachnospiraceae_NK4B4_group*, and *Christenseellaceae_R_7_group* were the dominant flora in the CdCl_2_ group. The criteria were LDA ≥3 and *p* < 0.05. In blood, *Acinetobacter* and *Bacillus* were the dominant flora in the control group, and *Muribaculaceae*, *Corynebacterium* and *Atopostipes* were the dominant flora in the CdCl_2_ group. With LDA ≥4.0 and *p* < 0.05 as the criteria. The results showed that the gut microbiota significantly differed from the characteristic flora in the blood in both the control and CdCl_2_ groups (Figure 4).

### 3.5. Effect of cadmium chloride on the serum metabolome

**Figure 5:**
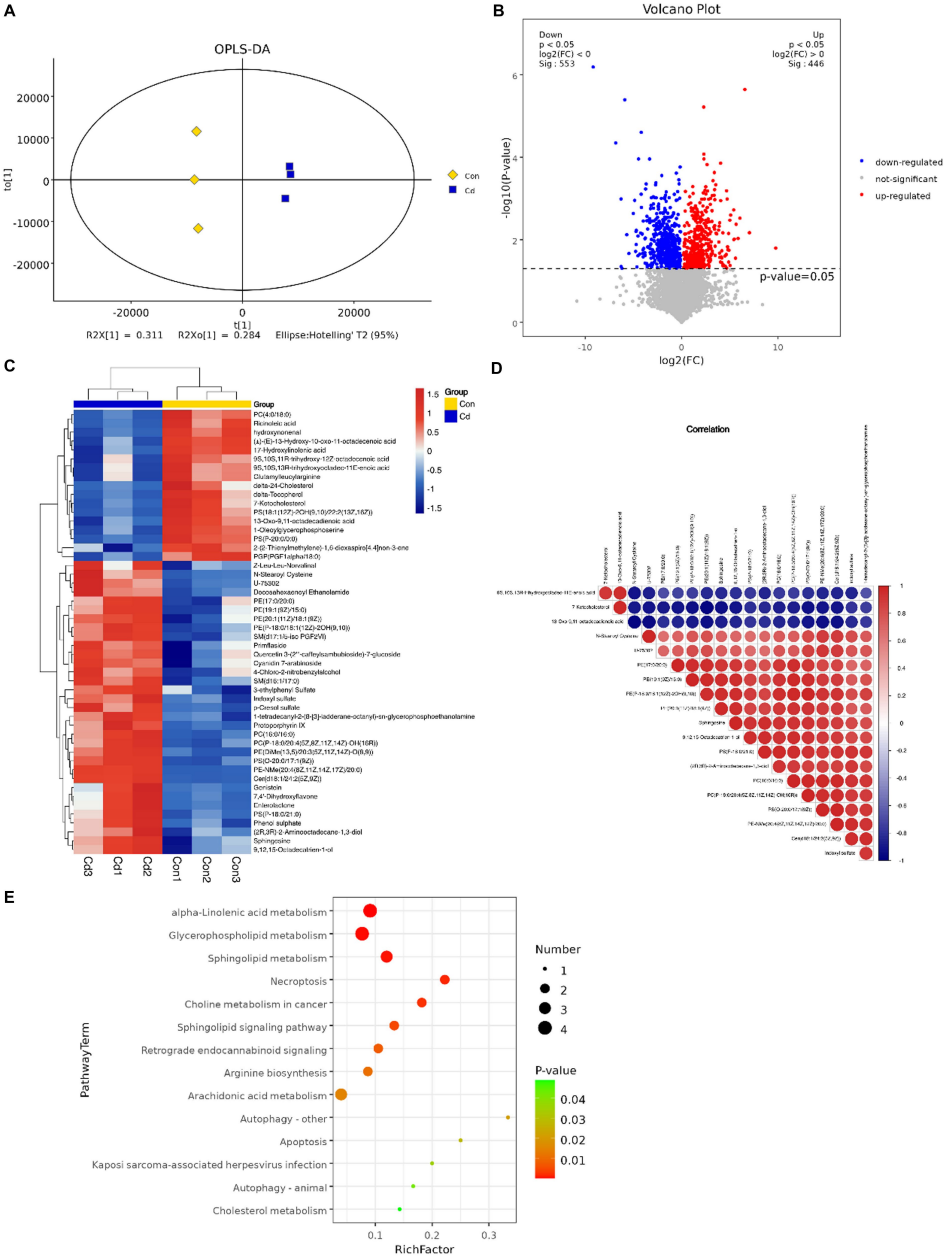
Multivariate statistical analysis of serum metabolites. **(A)** OPLS-DA score plot of serum metabolites. **(B)** Volcano plot of differential metabolites. Red dots represent significantly upregulated and blue dots represent significantly downregulated metabolites, whereas gray dots represent metabolites with no significant difference. Red dots indicate *p* < 0.05 and FC>1, blue dots indicate p < 0.05 and FC < 1. **(C)** The top 50 hierarchical clustering results of serum differential metabolite enrichment. Red and blue colors represent higher and lower concentrations of metabolites, respectively. **(D)** Pearson’s correlation coefficient was used to measure the correlation of metabolites and significant differences between the two groups. **(E)** KEGG pathway enrichment analysis results of differential metabolites (*p* < 0.05).

An OPLS-DA model revealed that serum metabolome in control and Cd exposure groups was distinct and was aggregated according to the treatment groups, with significant differences (R2Y = 0.998, Q2 = 0.876, Figure 5A). We conducted univariate statistical analysis to calculate the differences of metabolites detected in positive and negative ion modes. The resulting volcano plot effectively visualizes both p and FC values (Figure 5B). Furthermore, the metabolites were compared between the two groups by *t*-test, multivariate analysis, and influence on predicted variables. A total of 233 differential metabolites were identified and 22 potential metabolic biomarkers were screened (VIP >1.0; *p* < 0.05). Among the 22 biomarkers, 12 were upregulated and 10 were downregulated (Table 1). Hierarchical clustering of all significantly different metabolites and the top 50 significantly different metabolites ranked by variable important in projection (VIP) demonstrated that serum metabolic characteristics in the CdCl_2_ group changed significantly (Figure 5C). The metabolite correlation analysis showed significant interactions among metabolites. For example, 7-ketocholesterol was negatively correlated with indoxyl sulfate and *sphingosine* (Figure 5D). In addition, metabolites changed in rats after Cd exposure, including alpha-linolenic acid metabolism, glycerophospholipid metabolism, sphingolipid metabolism, necroptosis, and choline metabolism in cancer and other several other metabolic pathways (Figure 5E).

**Table 1:** The list of serum metabolites with significant changes in the CdCl_2_ group compared with the control group.

Metabolites	Sub class	VIP	*p*-value	FC
Sphingosine↑	Amines	10.140	0.023	0.587
Phenol sulphate↑	Arylsulfates	4.053	0.013	0.329
Indoxyl sulfate↑	Arylsulfates	7.339	0.007	0.373
p-cresol sulfate↑	Arylsulfates	4.322	0.003	0.313
Indole-3-acetylglycine↑	Amino acids, peptides, and analogs	1.545	0.022	0.318
Asparaginyl-proline↑	Amino acids, peptides, and analogs	2.297	0.035	0.356
N-stearoyl cysteine↑	Amino acids, peptides, and analogs	9.669	0.015	0.384
Phenylacetylglycine↑	Amino acids, peptides, and analogs	2.188	0.008	0.410
4-guanidinobutanoic acid↑	Amino acids, peptides, and analogs	1.424	0.037	0.545
Z-leu-leu-norvalinal↑	Amino acids, peptides, and analogs	6.335	0.034	0.726
Citrulline↑	Amino acids, peptides, and analogs	2.069	0.044	0.762
Asparaginyl-methionine↑	Amino acids, peptides, and analogs	1.547	0.026	0.785
L-glutamine↓	Amino acids, peptides, and analogs	1.093	0.030	1.491
Threonylglutamic acid↓	Amino acids, peptides, and analogs	1.584	0.020	1.867
Glutamylleucylarginine↓	Amino acids, peptides, and analogs	1.793	0.041	2.269
Docosahexaenoyl ethanolamide↓	Amino acids, peptides, and analogs	4.497	0.043	2.740
13-oxo-9,11-octadecadienoic acid↓	Lineolic acids and derivatives	7.017	0.007	2.568
Corchorifatty acid↓	Lineolic acids and derivatives	1.4236	0.008	2.618
F17-hydroxylinolenic acid↓	Lineolic acids and derivatives	3.893	0.003	3.103
(±)-(E)-13-hydroxy-10-oxo-11-octadecenoic acid↓	Lineolic acids and derivatives	5.870	0.003	3.622
7-ketocholesterol↓	Cholestane steroids	15.541	0.013	4.708
Delta-tocopherol↓	Quinone and hydroquinone lipids	4.765	0.006	11.818

### 3.6. Correlation between blood, gut microbiome and serum metabolites

**Figure 6:**
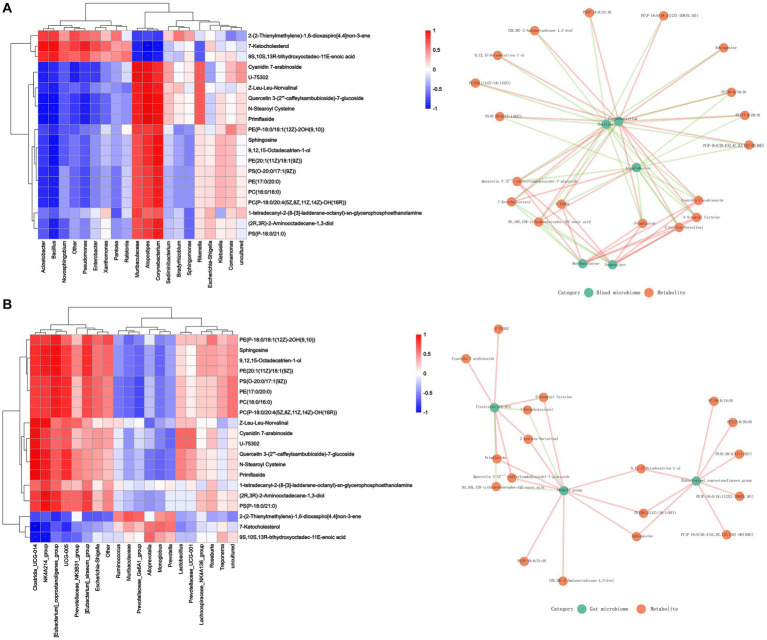
Effect of Cd exposure on microbial abundance and serum metabolite concentrations. **(A)** Correlation analysis between blood microbiome and metabolites. **(B)** Correlation analysis between gut microbiome and metabolites. In the heatmap, red indicates a positive correlation, whereas blue indicates a negative correlation. The darker color, is associated with a higher correlation. The closeness of the color to white represented a correlation closer to zero. ^***^p < 0.001, ^**^p < 0.01, and ^*^p < 0.05. In the correlation network diagram, green represents microorganisms and orange represents metabolites. The green line indicates a negative correlation, and the orange line indicates a positive correlation.

The correlation between microbial diversity and metabolomics was analyzed with Spearman’s correlation to better understand the microbial composition and functions. Sphingosine was found to be positively associated with the changes in the abundance of blood microbiome and included *Corynebacterium*, *Muribaculaceae*, and *Atopostipes* and gut microbiota including *Clostridia_UCG_014* and *NK4A214_group*. However, 7-ketocholesterol and 9S,10S,13R-trihydroxyoctadec-11E-enoic acid were negatively associated with these changes (Figure 6).

## 4. Discussion

After entering the body through the respiratory and digestive tracts, Cd tends to accumulate in different organs, resulting in problems including kidney injury and reproductive dysfunction (17). In addition, Cd exposure disrupts the intestinal flora, impairs host defense and homeostasis repair activities against pathogenic microorganisms, promotes inflammation in other organs, and increases disease susceptibility (26). Research conducted using antibiotic intervention and fecal transplantation has implicated gut microbiota in Cd exposure (17, 27). Our results demonstrated that the relative abundance of harmful bacteria, such as *Clostridia_UCG_014*, increased after Cd exposure. *Clostridia_UCG_014* is upregulated in T2DM rats and is positively correlated with blood glucose concentration; it is a potential proinflammatory bacterium (28, 29). However, the mechanisms that trigger toxicity *in vivo* remain largely understudied. The intestine is one of the targets for Cd (13) and its exposure may induce intestinal inflammation (9, 26), and increase the levels of inflammatory factors such as IL-6 and TNF-α in the blood and intestine. The correlation analysis revealed that the *Clostridia_UCG_014* and *NK4A214_groups* were significantly and positively correlated with TNF-α in the intestine, and the altered composition of this microbiota could be related to the occurrence of intestinal inflammation. In addition, Cd exposure could increase intestinal permeability, thereby disrupting the intestinal barrier and exacerbating intestinal damage (18). In addition, decreased expression of intestinal tight junction protein (ZO-1) induced by Cd exposure (9, 19) was confirmed in our experiment. ZO-1 contributes to the repair of intestinal mucosal injury (19), and its reduced activity is related to enhanced intestinal permeability (13, 18).

However, the breakdown of the intestinal barrier increases its permeability, allowing the introduction of intestinal flora and endotoxins into the blood circulation (30, 31), which can change the structure of blood flora, thereby affecting the physiological and pathological states of the body. Although dysregulation of the blood microbiome is intricately associated with the development of diseases (12), there exist no studies showing changes in the composition of the blood microbiome following Cd exposure. *Corynebacterium* is considered one of the microorganisms associated with bacteremia (32). We found a significant change in the abundance of *Corynebacterium* in intestinal and blood microbiome; especially, its abundance decreased significantly in the gut after Cd exposure but increased significantly in the blood. Whether Cd exposure can increase the risk of bacteremia warrants further investigation. The abundance of *Muribaculaceae* increased significantly after Cd exposure. However, *Muribaculaceae* was a characteristic flora in the gut of the high-fat diet group (33). Moreover, previous studies have reported that Cd exposure increased the risk of obesity (34). Moreover, the energy source of *Muribaculaceae* is primarily mucin-monosaccharide (35). Mucins are cell surface or secreted glycoproteins that contribute to the formation of mucosal barriers within the gut (36). *Muribaculaceae* has been implicated in mucosal layer degradation (37) by depleting intestinal mucus and thereby increasing intestinal barrier permeability. In conclusion, our results suggest that Cd-exposed blood bacteria could be characterized by an enhanced abundance of *Corynebacterium* and *Muribaculaceae*.

Cd exposure leads to changes in metabolic levels (38), and toxicometabolomics is expected to identify pathways and potential biomarkers of Cd toxicity (19, 39). Pathway enrichment analysis showed that Cd exposure largely affected lipid metabolism and α-linolenic acid metabolism; moreover, lipid metabolism has been demonstrated to be related to Cd exposure (17). Alpha-linolenic acid can restore high-fat diet-induced changes in the intestinal flora, improve intestinal barrier integrity, and inhibit inflammation (40). We found that alpha-linolenic acid metabolism was down-regulated after Cd exposure. We simultaneously screened four significant changes in serum metabolic biomarkers, namely, indoxyl sulfate (IS), phenyl sulfate, p-cresol sulfate, and δ-tocopherol. Previous studies have demonstrated that Cd exposure is closely related to the generation of oxidative stress (2). IS, phenyl sulfate, and p-cresol sulfate are uremic toxins produced by gut bacteria, and are associated with the development of CKD, reduce glutathione levels, and increase the vulnerability of the cells to oxidative stress (41, 42). Vitamin E protects against Cd toxicity and reduces oxidative damage (43). δ-Tocopherol, one of the major forms of vitamin E, inhibits the formation of oxidative and inflammatory mediators and can inhibit PhIP/DSD-induced colon cancer by preventing early cell and DNA damage (44). In addition, Cd exposure aggravates the damage to the intestinal barrier regulating L-glutamine and IS. L-glutamine is one of the primary energy sources in the gastrointestinal tract (45, 46). Depletion of L-glutamine during an infection or disease causes intestinal epithelial cell atrophy and hyperpermeability, whereas its supplementation alleviates intestinal permeability in patients with postinfectious irritable bowel syndrome (47). IS has been reported to induce intestinal barrier damage (48). Changes in the metabolic levels indicated that Cd exposure is associated with adverse physiological characteristics. We further explored the relationship between microbiota changes and metabolites and the results showed that *Corynebacterium*, *Muribaculaceae*, and *Atopostipes* were significantly and positively correlated with N-stearoyl cysteine, primflaside, quercetin 3-(2‴-caffeylsambubioside)-7-glucoside, Z-leu-leu-norvalinal. Therefore, changes in blood microbiome could be intricately related to metabolic changes.

## 5. Conclusion

In conclusion, we studied the changes in the composition of blood microbiome following Cd exposure. The results suggested that intestinal flora shift could be the mechanism underlying Cd-induced peripheral organ toxicity. Although there are many studies on the effects of Cd exposure on microbial communities, however，the relationship between translocation-induced changes in the composition of microbiome in the blood and metabolic changes remains unclear. Spearman’s correlation analysis revealed a correlation between the change in the flora abundance and the expression of metabolites and inflammatory factors. However, this study had certain shortcomings. For instance, the effect of blood microbiome dysregulation on metabolism-related diseases under Cd exposure could not be clarified. Therefore, future studies will focus on uncovering the causal relationship between blood microbiome and metabolic changes following Cd exposure, providing a basis for designing potential intervention approaches against Cd toxicity.

## Data availability statement

The datasets presented in this study can be found in online repositories. The names of the repository/repositories and accession number(s) can be found below: Sequence Read Archive of the National Center for Biotechnology Information repository; PRJNA989743.

## Ethics statement

The animal study was reviewed and approved by The Ethics Committee of Chengdu Normal University.

## Author contributions

BH and SL: conceptualization, funding acquisition, resources, and validation. JZ, BH, and SL: data curation and writing—review and editing. XD and WZ: formal analysis. ZL, GW, and BH: investigation. ZL, XD, and GW: methodology. ZL, WZ, and XD: software. BH, XD, and ZL: roles and writing—original draft. All authors contributed to the article and approved the submitted version.

## Funding

This work was supported by the Chengdu Normal University Scientific Research Project [No. 111-153701] (Acceptor: BH), the Agricultural Ecology and Green Food Development Project [CSCXTD2020B11] (Acceptor: SL), and National Scholarship Fund of China (grant no. 202208515063).

## Conflict of interest

The authors declare that the research was conducted in the absence of any commercial or financial relationships that could be construed as a potential conflict of interest.

## Publisher’s note

All claims expressed in this article are solely those of the authors and do not necessarily represent those of their affiliated organizations, or those of the publisher, the editors and the reviewers. Any product that may be evaluated in this article, or claim that may be made by its manufacturer, is not guaranteed or endorsed by the publisher.
